# Adiponectin, Leptin, and Resistin in Asthma: Basic Mechanisms through Population Studies

**DOI:** 10.1155/2013/785835

**Published:** 2013-10-30

**Authors:** Akshay Sood, Stephanie A. Shore

**Affiliations:** ^1^School of Medicine, Department of Medicine, University of New Mexico, MSC 10 5550, Albuquerque, NM 87131, USA; ^2^Department of Environmental Health, Molecular and Integrative Physiological Sciences Program, Harvard School of Public Health, 665 Huntington Avenue, Building I, Room 307, Boston, MA 02115, USA

## Abstract

Adipokines, factors produced by adipose tissue, may be proinflammatory (such as leptin and resistin) or anti-inflammatory (such as adiponectin). Effects of these adipokines on the lungs have the potential to evoke or exacerbate asthma. This review summarizes basic mechanistic data through population-based and clinical studies addressing the potential role of adipokines in asthma. Augmenting circulating concentrations of adiponectin attenuates allergic airway inflammation and airway hyperresponsiveness in mice. Murine data is supported by human data that suggest that low serum adiponectin is associated with greater risk for asthma among women and peripubertal girls. Further, higher serum total adiponectin may be associated with lower clinical asthma severity among children and women with asthma. In contrast, exogenous administration of leptin results in augmented allergic airway hyperresponsiveness in mice. Alveolar macrophages obtained from obese asthmatics are uniquely sensitive to leptin in terms of their potential to augment inflammation. Consistent with this basic mechanistic data, epidemiologic studies demonstrate that higher serum leptin is associated with greater asthma prevalence and/or severity and that these associations may be stronger among women, postpubertal girls, and prepubertal boys. The role of adipokines in asthma is still evolving, and it is not currently known whether modulation of adipokines may be helpful in asthma prevention or treatment.

## 1. Introduction

Obesity is increasingly appreciated as a risk factor for asthma and has been the subject of multiple recent reviews in the literature [1–3]. There is an immense interest in the potential role of adipose tissue in the development or worsening of asthma among obese individuals, particularly women. Adipose tissue is an active endocrine organ, producing numerous energy regulating hormones including adiponectin, leptin, and resistin. In obesity, serum concentrations of leptin and resistin increase, while adiponectin decreases. The adipose tissue of obese individuals is infiltrated with activated macrophages. Current concepts suggest that, during development of obesity, adipose tissue hypertrophy leads to local tissue hypoxia, focal adipocyte necrosis, and consequent recruitment of macrophages [4, 5]. Tissue hypoxia and macrophage activation, likely consequent to toll-like receptor (TLR) signalling *via* fatty acids, then result in the generation and release of a variety of proinflammatory cytokines, chemokines, acute phase proteins, and other moieties from adipose tissue. Collectively with adipose tissue derived hormones, these substances are referred to as adipokines. Serum concentrations of many of these adipokines are not only associated with body mass index (BMI) but are likely also mechanistically related to many obesity dependent diseases such as type II diabetes mellitus, steatohepatitis, atherosclerotic cardiovascular disease, and hypertension. Here, we consider the role of adipokines in obesity-related asthma. In particular, this review summarizes basic mechanistic data through population-based and clinical studies addressing the hypothesis that adiponectin, leptin, and possibly resistin may each have a role in asthma. The association between obesity and asthma is complex and multifaceted and is likely explained by many mechanisms, one of which involves these adipokines. 

## 2. Adiponectin 

Adiponectin is an insulin sensitizing hormone that also plays a role in inflammation. Adiponectin inhibits effects of proinflammatory cytokines, such as tumor necrosis factor (TNF)-*α* and interleukin-6 (IL-6), on endothelial and other cell types [6–8] and also induces expression of anti-inflammatory cytokines (IL-10 and IL-1 receptor antagonist) [8–10]. However, adiponectin also has proinflammatory effects that become manifest under selected conditions. For example, adiponectin induces IL-6 and matrix metalloproteinase-1 secretion in the synovial tissue of patients with arthritis [11]. Adiponectin can also induce activation of the proinflammatory transcription factor, nuclear factor (NF)-*κ*B in monocytic cell lines [12], while inhibiting the same in endothelial cells [13]. 

Curiously, even though visceral adipocytes are its most important source [14], systemic adiponectin concentrations are reduced in obesity [15]. Hyperinsulinemia, a common consequence of obesity, as well as excess proinflammatory cytokines (such as TNF-*α* and IL-6) that are produced in obese adipose tissue, has been shown to inhibit adiponectin mRNA expression in adipocytes and may contribute to obesity-related reduction in systemic adiponectin concentrations [16–18]. Further, incomplete processing of mature adiponectin protein, likely as a result of endoplasmic reticulum stress, has been proposed to play a role in the decline in serum adiponectin observed in obesity [19].

**Figure 1:**
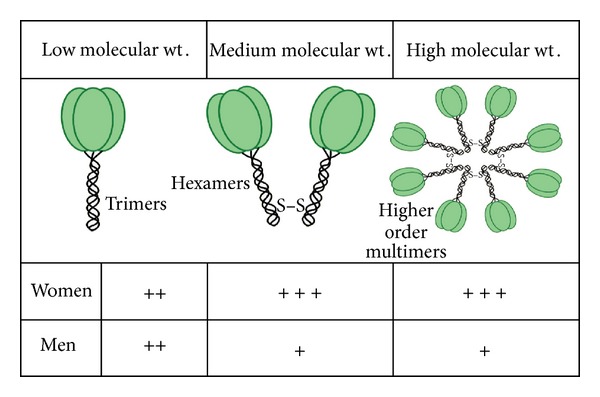
Schematic representation of the sexual dimorphism of the absolute concentrations of the circulating adiponectin isoforms. Compared to men, women have higher absolute concentrations of circulating total adiponectin (mean values of 11.3 versus 23.5 *μ*g/mL) and all its isoforms [26]. When the isoforms are expressed as a proportion of the total, women have higher proportions of high (32% versus 26%) and medium molecular weight (39% versus 33%) isoforms but a lower proportion of the low molecular weight isoform (28% versus 41%) than men [26]. The figure summarizes the data published previously by Peake et al. [26] and has been reproduced with permission from Curr Med Chem [27].

Adiponectin monomers have a globular head and a collagen-like tail. However, adiponectin monomers do not circulate. Instead, adiponectin multimerizes and circulates in the blood as trimeric, hexameric, and higher order multimeric forms that have low, medium, and high molecular weights (LMW, MMW, and HMW), respectively, as shown in Figure 1. A globular form can also be created at target organs by proteolytic cleavage of the collagenous tail. Although this isoform is biologically active, it is not clear that it exists in the circulation. There are gender differences in both the serum concentration and isoform distribution of adiponectin (Figure 1). Total serum adiponectin and particularly the HMW isoform, is higher among women than men [20]. These differences develop during puberty and are the result of inhibition of HMW adiponectin production by circulating testosterone [21]. While the LMW and HMW isoforms of adiponectin dominate in the serum of men and women, respectively [22], the MMW isoform appears to dominate in the sputum without any sex-related difference in concentrations [23]. MMW and HMW adiponectin isoforms also dominate in the murine lung lining fluid [24]. Overall, there is poor correlation between blood and airway concentrations of total adiponectin or its isoform distribution [23, 25] likely because in the absence of inflammation adiponectin does not easily cross the pulmonary vasculature to access the lung (see the following). 

Adiponectin isoforms vary in efficacy. For example, HMW adiponectin is the most biologically active isoform with respect to insulin sensitivity [28, 29]. Similarly, HMW adiponectin correlates inversely with cardiovascular risk factors (triglycerides, hypertension) and positively with cardioprotective factors (high-density lipoprotein or HDL cholesterol), even after controlling for BMI, whereas such correlations are not observed for other isoforms of adiponectin [30]. The activity of the various isoforms with respect to their effects on the airway remains to be determined. 

Several adiponectin binding proteins have been identified, including AdipoR1, AdipoR2, T-cadherin, and calreticulin. Adiponectin can also induce effects in a receptor-independent fashion. Multiple cell types in the lung express adiponectin binding proteins, including the bronchial epithelium [31, 32], airway smooth muscle [33], and pulmonary vasculature [34, 35]. Expression of AdipoR2 and T-cadherin mRNA by bronchial epithelial cells is greater among obese patients with asthma than obese controls [36]. Adiponectin binding proteins differ in their affinity for the various adiponectin isoforms. Globular adiponectin primarily binds to AdipoR1, whereas full-length (multimeric) adiponectin primarily binds to AdipoR2 [37]. AdipoR1 and AdipoR2 have been shown to result in peroxisome proliferator-activated receptor (PPAR)-*α*, adenosine monophosphate-activated protein kinase (AMPK), and p38 mitogen-activated protein kinase (MAPK) activation [37, 38]. AdipoR1 and AdipoR2 exhibit ceramidase activity, and adiponectin augments ceramide conversion to sphingosine-1-phosphate (S1P). In fact, adiponectin induced AMPK activation may be mediated through the actions of S1P. The ability of adiponectin to inhibit apoptosis in cardiomyocytes and other cells may be the result of this ability to convert the proapoptotic ceramide to the antiapoptotic S1P [39]. Because several S1P receptors with diverse effects exist, it is conceivable that the seemingly contradictory pro- and anti-inflammatory effects of adiponectin result from stimulus and cell-type specific differences in the expression of S1P receptors. It has not been determined whether adiponectin couples to ceramidase activity in the lungs, but adiponectin does inhibit apoptosis in bronchial epithelial cells [32], and loss of such antiapoptotic effects may explain why adiponectin deficient mice develop emphysema-like changes as they age [40]. Moreover, adiponectin induced effects in bronchial epithelial cells include augmented wound healing and proliferation as well as IL-8 release [31, 32]. The airways are therefore a likely target of adiponectin.

T-cadherin appears to be important for adiponectin transport into the lungs. The concentration of adiponectin in murine bronchoalveolar lavage (BAL) fluid is relatively high [31, 40]. Because adiponectin is not synthesized to any physiologically meaningful extent in the murine lung [40], it must be taken up into the lung from the blood. However, adiponectin does not enter the lung *via* simple diffusion through gaps between endothelial cells. HMW adiponectin dominates in BAL fluid in mice, with lesser amounts of MMW adiponectin and very little LMW adiponectin [24, 34], a pattern opposite to that expected by diffusion. T-cadherin is abundantly expressed on endothelial cells and avidly binds HMW and MMW but not LMW adiponectin [41, 42]. Furthermore, adiponectin is lower in BAL fluid of otherwise naïve T-cadherin deficient versus wild-type mice. These data are consistent with the hypothesis that adiponectin binding to T-cadherin on pulmonary vascular endothelial cells permits adiponectin transit across these cells by a vesicular transcytosis pathway in mice. Adiponectin bound to T-cadherin on endothelial cells also appears to serve as a repository for adiponectin, since serum adiponectin is markedly elevated in T-cadherin deficient versus wild-type mice, and the marked adiponectin immunoreactivity normally present on endothelial cells is absent in T-cadherin deficient mice [24, 34, 41]. It is important to note that whereas T-cadherin appears to be important for transit of adiponectin from blood to lungs in naïve mice, this is not the case in mice with pulmonary inflammation/injury sufficient to increase alveolar capillary permeability. In both allergen exposed and ozone exposed mice which have marked increases in BAL protein consistent with leak of serum proteins into the alveolar spaces, there is also a marked increase in BAL adiponectin [43, 44]. Moreover, under these circumstances, T-cadherin deficiency no longer results in reductions in BAL adiponectin, and BAL fluid becomes dominated by LMW adiponectin [44], consistent with diffusion of adiponectin through paracellular pathways between endothelial cells. 

### 2.1. Adiponectin and Asthma

Given the presence of adiponectin and its receptors in the lung and the declines in adiponectin concentrations in obesity, it is conceivable that the loss of the anti-inflammatory effects of adiponectin in obesity contributes to asthma prevalence or severity. Below, we discuss data exploring this hypothesis. We first discuss studies performed in animals and then present data from population-based and clinical studies in humans.

#### 2.1.1. Animal Studies

**Figure 2:**
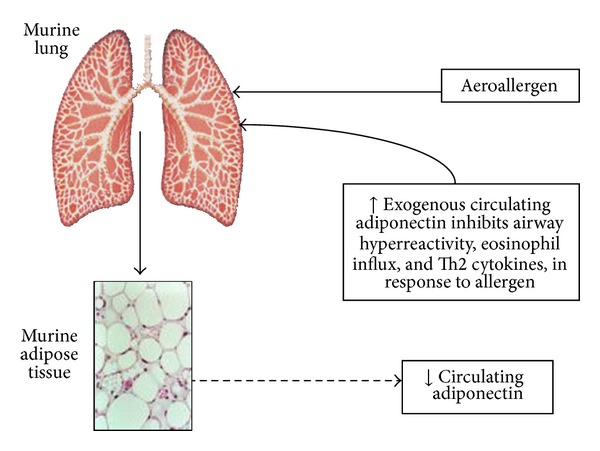
A schematic representation of the suggested role for adiponectin in allergen induced asthma in mice, based upon the work by Shore et al. [46]. This figure is as originally published by Sood et al. [49].

In mice, obesity-related declines in adiponectin appear to contribute to the development of type 2 diabetes mellitus and atherosclerosis. Exogenous administration of adiponectin protects obese mice against these conditions, while adiponectin knockout mice are susceptible (see [45] for review). Shore et al. [46] sought to determine whether the effects of adiponectin in the lung are consistent with a possible role for this adipokine in asthma. To do so, they administered exogenous adiponectin to lean allergen sensitized mice during acute allergen challenge. Mini-Alzet pumps were used to allow for continuous infusion of adiponectin throughout the challenge period and resulted in an approximate 50% increase in serum adiponectin. Compared to vehicle, adiponectin treatment resulted in a marked reduction in allergen induced airway hyperresponsiveness (Figure 2). Adiponectin treatment also caused an almost complete suppression of eosinophil recruitment to the airways and of Th2 cytokine expression in the lungs. Ionescu et al. [47] obtained a similar result in a murine model of *chronic* allergen challenge. They showed that intranasal administration of adiponectin along with each allergen challenge resulted in a marked suppression of allergen induced airway hyperresponsiveness and airway inflammation, and also inhibited the increases in airway smooth muscle thickness that are observed in this model. Adiponectin also reduces the platelet-derived growth factor (PDGF) or serotonin induced proliferation of murine airway smooth muscle cells in culture (unpublished observations), consistent with its inhibitory effects on vascular smooth muscle proliferation [35]. Interestingly, in rat vascular smooth muscle, the antiproliferative effects of adiponectin are observed with the globular but not the full-length isoforms, suggesting that proteolytic cleavage of this adipokine is required for its biological effects on smooth muscle [48]. 

Despite the marked reductions in allergic airways responses observed with adiponectin treatment after acute allergen challenge in sensitized mice [46], adiponectin deficiency does not augment these responses in this model [43, 50], although it does augment responses to *chronic* allergen challenge [50]. It is conceivable that the differential effects of adiponectin deficiency in acute versus chronic allergen challenge models relate to the effects of the challenge on endogenous adiponectin. Shore et al. [46] reported a reduction in both serum adiponectin and in adipose tissue adiponectin mRNA expression in mice after acute allergen challenge, presumably as a result of spillover of inflammatory moieties from the airways to the blood. Such reductions would limit differences between wild-type and adiponectin deficient mice. In contrast, the chronic allergen challenge model used [50] results in a much less intense inflammation and presumably less extensive reduction in serum adiponectin. 

Studies addressing the nature of the receptors that contribute to the protective effect of adiponectin for asthma on the lung are more limited. Williams et al. observed a marked reduction in allergen induced airway hyperresponsiveness, eosinophil recruitment to the airways, Th2 cytokine expression, and mucous cell hyperplasia in mice deficient in T-cadherin [43]. Importantly, these reduced responses to allergen were not observed in mice that were bideficient in both T-cadherin and adiponectin, indicating that adiponectin was required for the effects of T-cadherin deficiency to be manifest and therefore that the adiponectin binding properties of T-cadherin were involved in the observed responses. The data indicate that T-cadherin does not mediate the ability of adiponectin to reduce allergic airways responses. Instead, the effects of T-cadherin deficiency were likely secondary to the increased serum concentrations of adiponectin observed in these mice. 

The cell type that is the target of effects of adiponectin that limit allergic airway responses has also not been established. In addition to epithelial cells, endothelial cells, and airway smooth muscle (see above), it is conceivable that lung macrophages are involved. Eotaxin, an important eosinophil chemotactic factor, is released from cultured bone marrow derived macrophages treated with IL-4 and TNF, and adiponectin attenuates this release [50]. Others have also reported that adiponectin biases macrophages towards an M2, less inflammatory phenotype [51], though there are also reports that adiponectin increases the inflammatory potential of human macrophages [52]. Dendritic cells undergo functional maturation in response to adiponectin [53], and T cells do express adiponectin receptors [54], but adiponectin does not affect T cell proliferation after allergen presentation by dendritic cells [43, 52]. However, adiponectin does induce increased IFN-*γ* production by activated CD4+ T cells along with increased expression of the transcription factor, T-bet, indicating greater Th1 bias [52, 53]. Such Th1 biasing of CD4+ T cells might limit allergic airway responses that require Th2 cells. 

It is interesting to note that compared to wild-type mice, T-cadherin deficient mice with high serum adiponectin also had reductions in BAL IL-17A expression after allergen challenge [43]. Moreover, allergen induced increases in BAL IL-17 were restored when the T-cadherin deficient mice were also deficient in adiponectin [43]. The results indicate that adiponectin may regulate IL-17A expression in the lungs. Consistent with these observations, in mice challenged with ozone (0.3 ppm for 24–48), Kasahara et al. [44] observed a marked increase in IL-17A mRNA expression in adiponectin deficient versus wild-type mice. Both pulmonary interstitial macrophages and *γδ* T cells produced IL-17A after ozone exposure and may have been the targets of adiponectin. 

Despite the largely anti-inflammatory effects of adiponectin in the setting of allergic airways disease, adiponectin also appears capable of causing proinflammatory/proasthmatic effects in the lungs in response to other stimuli. Following high dose acute ozone exposure (2 ppm for 3 h), mice develop airway hyperreactivity and a neutrophilic inflammation characterized by increases in acute phase cytokines and chemokines. These responses are attenuated in mice deficient in adiponectin [24]. Nevertheless, compared to wild-type mice, responses to acute ozone exposure are unaffected in transgenic mice that substantially overexpress adiponectin [24]. These results suggest that normal endogenous levels of adiponectin are sufficient to produce maximal augmentation of proinflammatory effects of acute ozone exposure. Under such circumstances, further increasing of adiponectin by transgenic overexpression would not be expected to have any additional effects, as observed. 

#### 2.1.2. Human Studies

There are also data from human subjects suggesting a protective role for adiponectin in asthma. It is important to note that reagents necessary to perform mechanistic studies in humans are still lacking and awaiting a better understanding of adiponectin signalling processes. Hence, studies in human subjects have been mostly limited to associations between circulating or lung levels of adiponectin and clinical disease outcomes with limited data on inflammatory outcomes (Table 1). The assumption underlying such studies is that if adiponectin is mechanistically related to asthma, then there ought to be associations between disease prevalence or severity and adiponectin concentrations. In evaluating these studies, one must bear in mind that even in animal studies the target cell mediating the apparent beneficial effects of adiponectin has not been established. Therefore, it is not clear whether blood adiponectin, lung adiponectin, or the adiponectin concentration in some other target tissue, for example, the lymph nodes or perilymphatic fat tissue, is appropriate to examine. This is a particularly difficult issue given the lack of correlation between blood and lung adiponectin (see above). 

*(i) Asthma Prevalence*. Some but not all studies demonstrate that low serum total adiponectin concentrations are associated with a greater risk for asthma among women and peripubertal girls (see Table 1). 

The strongest evidence supporting a relationship between adiponectin and asthma comes from a US-based longitudinal cohort that showed that low serum total adiponectin concentrations (<7 mg/L) are associated with increased risk for incident asthma among women and that this association was significantly stronger among current smokers versus not currently smoking women [69]. Further, low serum total adiponectin was a stronger predictor than BMI for incident asthma among women [69]. Interestingly, the converse was not true, that is, prevalent asthma did not predict low future serum total adiponectin concentrations [69].

**Figure 3:**
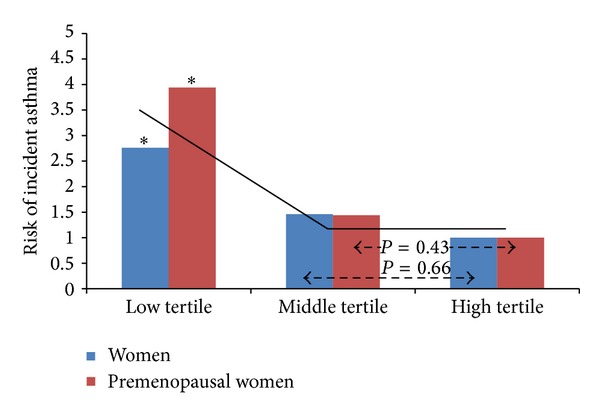
Nonlinear relationship between serum adiponectin and risk of incident asthma in a US-based longitudinal study [69]. The depicted relationship may show a threshold effect that is only seen with the lowest tertile of serum adiponectin concentration. The symbol * represents a significant (*P* < 0.05) comparison with respect to the high tertile. In another US-based cross-sectional study, a similar threshold effect was seen between the highest quartile of serum leptin concentration and prevalent asthma in women [66]. Reproduced with permission of the American Thoracic Society. Copyright © 2013 American Thoracic Society. Official Journal of the American Thoracic Society [69].

Unlike the above-mentioned longitudinal study [69], five relatively large human studies cross-sectionally analyzed the association between serum adiponectin and prevalent asthma, independent of obesity [55, 63–65, 67]. While three of these five studies showed no significant associations [55, 64, 65], two other studies showed that low serum total adiponectin was associated with greater odds for asthma among premenopausal women and peripubertal girls [63, 67]. Although the birth cohort study in New Zealand showed that serum total adiponectin concentrations were not associated with prevalent asthma diagnosis overall, sex-specific analyses showed that high serum total adiponectin among men was associated with lower exhaled nitric oxide levels but confusingly greater odds for prevalent bronchodilator responsiveness [65]. Thus, the spirometric data and exhaled nitric oxide data show contradicting physiologic and anti-inflammatory effects of adiponectin among men [65]. Generally speaking, many of the above-mentioned cross-sectional analyses were limited by their smaller numbers of girls/women, modest effect sizes, and lower prevalence of asthma and obesity in populations outside the United States [55, 64, 65]. Compared to the inconclusive adiponectin-asthma studies [55, 64, 65], the US-based longitudinal cohort [69] possibly had a greater statistical power due to both a planned selection of large numbers of African Americans as well as a fortuitous selection of large numbers of obese subjects and smokers—these population groups are known to be particularly associated with low serum total adiponectin concentrations [14]. The longitudinal study further found a nonlinear relationship between serum total adiponectin and risk for incident asthma (Figure 3) [69]. Since the relationship may show a threshold effect that is only seen with the lowest tertile of serum adiponectin concentration (at <7 mg/L in that study), it is important to have adequate numbers in this group for any study to demonstrate a significant effect on incident asthma. This may explain the discrepant results from other studies in this field [55, 64, 65, 69]. 

Another explanation for these apparent discrepancies is that serum concentration of adiponectin may be a poor surrogate for the adiponectin that actually impacts asthma. In a preliminary study by Sood et al. on nonsmokers, sputum total adiponectin concentrations were lower among asthmatics than controls [23]. In fact, low sputum adiponectin predicted asthma status better than other measures of adiposity including serum adiponectin, serum leptin, BMI, or DEXA measures of fat and lean mass in that study [23]. On the other hand, several other small case-control studies failed to find any difference in BAL adiponectin concentrations between asthmatics and controls, after matching or adjusting for obesity [25, 70–72]. One explanation for the discrepant findings between these studies is that sputum adiponectin may be a relatively stronger measure than BAL adiponectin for examining this association [23, 25, 70–72], since the former is approximately 40 times more concentrated than the latter.

Although it is certainly possible that adiponectin does not modify asthma in humans, another potential explanation for discrepant findings between studies lies in the possible nonlinear effects of adiponectin that are missed by linear statistical modelling. These effects may be threshold effects (i.e., manifest only when a critical threshold in serum concentrations is reached, as suggested by Sood et al. [69]) or U-shaped effects (with associations apparent at both extremes of serum concentrations). 

*(ii) Asthma Severity*. Data here are limited, but higher serum total adiponectin concentrations may be associated with lower clinical asthma severity among children (particularly boys) and women with asthma but interestingly greater disease severity among men (Table 1). Unlike clinical severity measures, adiponectin is not associated with inflammatory asthma measures in a limited number of studies.

In a large community-based cross-sectional study of subjects with asthma, Sood et al. showed that high serum total adiponectin was associated with more frequent active disease (including more frequent use of any asthma medication) and greater number of respiratory symptoms and asthma medications among men with asthma but beneficial effects among women with asthma, with significant sex-specific interactions [49]. These findings in women [49] were consistent with those of another small study of postmenopausal women with asthma that showed that high serum total adiponectin was associated with milder clinical severity of disease (mean values 16.6 ng/mL in mild to moderate asthma versus 9.8 ng/mL in severe asthma); men with asthma were not included in that study [70]. Similarly, among postpubertal obese girls who underwent weight loss, a modest increase of 28% in baseline serum total adiponectin was associated with an increase in spirometric parameters and reduction in asthma severity in subjects with asthma—this analysis was however limited by inadequate adjustment for the confounding effect of decrease in BMI [68]. Unlike clinical and lung function measures, lung inflammatory biomarkers have not been shown to be associated with adiponectin (in either serum or bronchoalveolar lavage fluid) [25].

Pediatric studies, examining mostly prepubertal and peripubertal asthmatic boys, show a consistent trend. High serum total adiponectin concentrations were associated with less severe exercise induced bronchoconstriction [56] as well as fewer maximum asthma symptom days; fewer asthma exacerbations; higher FEV_1_/FVC ratio [62], and higher FEF_25–75%_ values [55]. 

In a small study of obese women undergoing bariatric surgery, adiponectin mRNA expression in visceral abdominal (omental) adipose tissue from asthmatics was lower than that from controls, even after adjustment for BMI, and even though no differences in serum adiponectin concentrations were reported between the two groups [36]. Thus, it may be that adipose tissue adiponectin mRNA expression is simply a marker for overall adipose tissue-dependent effects that impact asthma, perhaps through other aspects of the systemic inflammation of obesity. Such an explanation could explain why anti-inflammatory effects of serum adiponectin on asthma outcomes appear stronger in prepubertal boys than men. In prepubertal boys, testosterone effects on serum adiponectin are not yet fully manifest, so serum adiponectin is more closely representative of other obesity-related effects in the adipose tissue. T-cad genotype could also impact the fidelity of serum adiponectin in reporting adipose tissue dependent events. Reports from several large genome wide association studies indicate that variation in serum adiponectin concentrations among humans is in part explained by single-nucleotide polymorphisms (SNP) in the promoter of the T-cad gene [73, 74]. Given the marked increase in serum adiponectin induced by T-cadherin deficiency in mice [24, 43], along with observed reductions in pulmonary T-cadherin expression induced by allergic airways inflammation [43, 46], it is also possible that asthma itself interferes with the ability of serum adiponectin to report events occurring in adipose tissue. 

Another explanation for the data from the morbidly obese asthmatics undergoing bariatric surgery is that the change in adiponectin expression was a consequence and not a cause of asthmatic bronchoconstriction. Indeed, others have shown that acute severe asthma exacerbations result in a transient decrease in serum adiponectin concentrations [70], similar to the reductions observed in mice challenged with allergen [46], whereas bronchoprovocation from experimental inhalational allergen challenge does not acutely affect serum adiponectin concentrations [75], perhaps because the bronchoconstriction is so transient. Adipose tissue hypoxia is thought to be central to obesity-related adipose tissue inflammation [4], and it is likely that during severe asthma exacerbations, ventilation perfusion mismatch, and consequent hypoxemia would worsen this adipose tissue hypoxia and thus decrease adiponectin expression. Alternatively, systemic spillover of inflammatory moieties derived from the lung could impact adipocyte gene expression, as discussed above. While this spillover may not be significant in stable asthma [69] or transient asthma exacerbation [75], it may become manifest in either inadequately controlled asthma in morbidly obese subjects [36] or during a severe acute asthma exacerbation [70].

In summary, adiponectin is present in the lung, and adiponectin receptors are expressed on key lung cells. There are relatively strong data indicating that adiponectin limits allergic airways responses in lean mice, though in models of non allergic asthma, such as ozone exposure, adiponectin paradoxically promotes rather than inhibits airway hyperresponsiveness. Similarly, it is possible that proinflammatory airway effects of adiponectin dominate under certain physiologic conditions in humans, while anti-inflammatory airway effects dominate under others. For instance, in subjects with chronic obstructive pulmonary disease (COPD), an inflammatory obstructive airway disease related to asthma, proinflammatory airway effects of adiponectin appear to dominate [31, 76–79]. This may also be the case among men with asthma [49]. On the other hand, anti-inflammatory airway effects of adiponectin may dominate among women and children, although the literature is not consistent. Some of the lack of consistency of the human data may relate to the inadequacy of serum total adiponectin as a marker of the concentrations of relevant isoforms at target cells within the lung. It also remains possible that the observed associations are the result of the ability of serum adiponectin to report other events occurring in adipose tissue that impact asthma. Further understanding of the adiponectin target cells and signalling pathways might help establish whether adiponectin has a causal role in human asthma and further allow for therapeutic strategies that harvest the beneficial while limiting the deleterious effects of this adipokine. 

## 3. Leptin 

The hormone leptin, which derives its name from the Greek word leptos, meaning thin, is a 16 kD protein derived from the obese (ob) gene and is expressed predominantly in adipocytes [80]. Consequently, leptin is produced in proportion to adipocyte mass, and leptin concentrations are 4–6 times greater in severely obese compared to lean human subjects [81]. Serum leptin concentrations increase with feeding and in infectious and inflammatory states [82, 83]. Serum leptin concentrations also display a circadian rhythm, with a nadir around 8 in the morning [84]. Women and postpubertal girls have 40–200% higher concentrations of circulating leptin than their male counterparts, after adjustment for percent body fat [85–88]. The rate of increase of circulating leptin concentrations with BMI is about three-fold more rapid in women as in men [89]. Although estrogen supplementation increases and testosterone supplementation decreases leptin concentrations [86, 90], sex differences in circulating leptin are not entirely explained by either sex hormones or body fat distribution [87]. Instead, these sex differences may involve greater leptin gene expression in subcutaneous than visceral adipose tissue [91] (subcutaneous adipose tissue is more abundant in women) and/or greater release of leptin from nonadipose sources [92]. Leptin can be expressed by various cell types in the human lung, including bronchial epithelial cells and alveolar type II pneumocytes and macrophages [93, 94]. Whether these cells result in leptin production that is significant compared with that provided through the blood remains to be established. However, the observations that airway leptin concentrations (measured in either sputum or BAL fluid) are strongly correlated with serum leptin concentrations (*r* ^2^ of 0.61 and ≥0.55 for sputum and BAL fluid, resp.) [23, 25] suggest that pulmonary sources may be of limited physiological importance. Such correlations also suggest that, in contrast to adiponectin, leptin is readily transported from the blood to the lung.

Leptin mediates its effect by binding to the leptin receptor (OB-R), a single membrane spanning receptor of the class I cytokine family with closest homology to gp130 of the IL-6 family of cytokine receptors. Several OB-R variants are generated by alternative splicing. The intracellular domain of each of these, except OB-Re, which lacks the transmembrane domain and is a soluble receptor, contains an interaction motif for members of the janus kinase (JAK) family of tyrosine kinases. However, only the long form, OB-Rb, contains a binding motif for members of the signal transducer and activator of transcription (STAT) family of transcription factors [95]. Following leptin binding, homo-oligomerization of leptin receptors occurs. JAKs, which are constitutively associated with the receptors, become phosphorylated, and in turn tyrosine phosphorylates the receptor itself creating docking sites for SH2 domains in STATs. JAKs then phosphorylate the STATs leading to their nuclear translocation and to gene transcription. Receptor phosphorylation also leads to recruitment of adaptor proteins that result in activation of mitogen-activated protein kinase (MAPK), phosphatidylinositol 3-kinase (PI3K), and mammalian target of rapamycin (mTOR). AMPK is also activated by leptin [96]. Ob-Ra is expressed in the lung, where it accounts for the majority of Ob-R expression [97], although it is unclear whether the receptor isoforms, which lack the STAT binding domain, are capable of signalling *in vivo* [96]. The lung is also among the peripheral tissues with the highest OB-Rb expression [97]. Pulmonary and bronchial epithelial cells express leptin receptors [94, 98], as do airway smooth muscle cells [99]. 

Leptin plays a key role in the regulation of appetite, metabolism, and body weight, and both leptin deficient and OB-Rb deficient mice are massively obese [100, 101]. Leptin also has profound effects on both innate and adaptive immune system that may impact asthma. Leptin promotes neutrophil chemotaxis and generation of reactive oxygen species, induces activation of NK cells, and also promotes macrophage activation, phagocytosis, and cytokine release [102]. The ability of leptin to upregulate leukotriene biosynthesis in alveolar macrophages [103] may be particularly relevant to asthma given the potency of cysteinyl leukotrienes as bronchoconstrictors. The observation that leptin deficient mice have thymic atrophy [104] emphasizes the role of leptin in adaptive immunity. CD4+ T cells express OB-Rb, and leptin induces proliferation of naïve but not memory T cells [104]. Leptin also differentially affects cytokine production in Th1 and Th2 cells; leptin increases production of Th1 cytokines including interferon-*γ* but suppresses production of Th2 cytokines including IL-4 [104]. It is increasingly appreciated that IL-17 may contribute to severe asthma. It is interesting, in this context, that when purified naïve splenic CD4+ T cells are grown under Th17 biasing conditions, leptin administration augments Th17 cell generation [105]. Leptin also inhibits the function of regulatory T cells (Tregs) [106], which would be expected to augment allergen induced T cell activation. 

Effects of leptin on lung cells might also impact asthma. Bronchial epithelial cells are increasingly recognized as contributing to asthmatic airway inflammation. Bronchial epithelial cells express leptin receptors, and leptin causes epithelial cell proliferation and mucin protein expression [94, 98, 107]. Airway smooth muscle cells also express leptin receptors capable of STAT-3 phosphorylation, and leptin inhibits PDGF induced proliferation in these cells [99]. Leptin also promotes the ability of PDGF to induce VEGF release from airway smooth muscle cells [33] and reduces the ability of IL-13 stimulated airway smooth muscle cells to produce eotaxin but has no effect on airway smooth muscle cell contraction [99]. Leptin receptors are expressed in mast cells both in the lungs and other tissues [108], but the precise effects of leptin on pulmonary mast cells remain to be established. 

### 3.1. Leptin and Asthma

Given the effects of leptin on the immune system and the lung, it is conceivable that obesity-related increases in leptin could initiate or worsen asthma. Data from both animal and human studies that have explored this hypothesis are discussed below.

#### 3.1.1. Animal Studies

**Figure 4:**
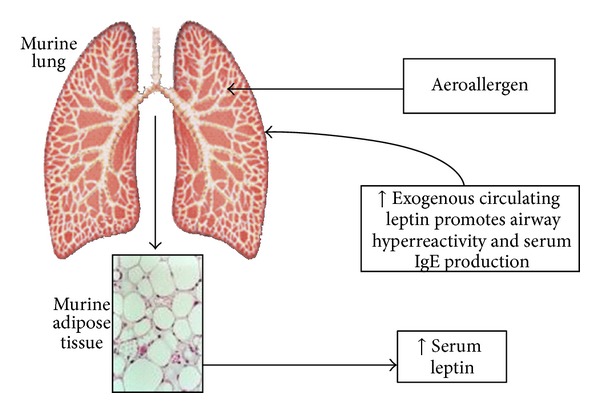
A schematic representation of the suggested role for leptin in allergen induced asthma in mice, based upon the work by Shore et al. [109]. Reproduced with permission from Biochimie [112].

Shore et al. [109] examined allergen sensitized and challenged mice in which leptin was experimentally increased by continuous infusion of leptin *via* mini-Alzet pumps. Compared with saline, leptin had no effect on airway responsiveness in unchallenged mice but increased allergen induced airway hyperresponsiveness (Figure 4). However, neither BAL eosinophils nor Th2 cytokines were affected by leptin treatment, suggesting that leptin dependent modifications in airway hyperresponsiveness were independent of effects on T cells. Although it is conceivable that leptin effects on mast cells could have played a role, especially since leptin treatment did result in increased serum IgE [109], the model used was not one that is mast cell dependent. Instead, effects of leptin on the innate immune system might have led to the observed increase in airway hyperresponsiveness. In lean mice, leptin treatment that results in serum leptin concentrations consistent with those observed in obesity, increases inflammatory responses to acute ozone exposure [100]. Such exposures result in TLR activation [110]. However, fasting, which substantially reduces serum leptin, had no effect on ozone induced inflammation, nor was ozone induced inflammation affected in fasted mice when serum leptin was returned to normal levels by exogenous administration [111]. Taken together, the data indicate that leptin induced augmentation of the inflammatory response to ozone requires increases in serum leptin above those normally observed in lean mice, that is, increases such as those observed in obesity. 

Leptin deficient (*ob/ob*) and leptin receptor deficient (*db/db*) mice each exhibit innate airway hyperreactivity [100, 101]. Arteaga-Solis et al. [113] recently reported that this airway hyperreactivity may be the result of loss of central effects of leptin that inhibit parasympathetic drive to airway smooth muscle cells, since the changes in pulmonary mechanics were alleviated in mice with parasympathetic blockade. Shore et al. [109] did not observe this apparent bronchodilatory effect after exogenous administration of leptin; in nonallergic mice, airway responsiveness was similar in mice treated with saline or leptin. However, it is conceivable that exogenous leptin was unable to cross the blood brain barrier to any substantive extent, limiting any central effects on parasympathetic drive. Nevertheless, it is unlikely that the innate airway hyperreactivity observed in *ob/ob* and *db/db* mice [100, 101] is solely the result of loss of bronchodilatory effects of leptin, since this airway hyperreactivity is also observed in other types of obese mice with elevated serum leptin [114, 115]. 

#### 3.1.2. Human Studies

Studies in human subjects have been mostly limited to associations between circulating or lung levels of leptin and clinical disease outcomes such as asthma prevalence or severity. Data on inflammatory outcomes is very limited. Further, since there are no longitudinal or interventional studies examining this association, the direction of causation for this association cannot be definitively established. Our understanding of the effect of leptin on asthma in humans, independent of the confounding effect of BMI, is therefore still evolving (see Table 1). 

*(i) Asthma Prevalence*. Some but not all studies indicate that high serum leptin concentrations are associated with greater odds for asthma prevalence, particularly among prepubertal boys, peripubertal and postpubertal girls, and women (Table 1). The data suggest that this association may be more consistent in children than in adults. 

#### 3.1.3. Population-Based Studies

A large cross-sectional analysis of participants of the US-based Third National Health and Nutrition Examination Survey (NHANES III) showed that women with high serum leptin concentrations (>21.9 ng/mL) had greater odds for asthma than those with low concentrations, independent of triceps skin fold thickness. This association was stronger among premenopausal women than postmenopausal women [66]. These findings were, however, not confirmed by Sutherland et al. in a population-based birth cohort of approximately 1,000 young adult New Zealanders [65] or by Jartti et al. [64] in a large sequential cross-sectional study set within an established Finnish cohort. There are various reasons why the findings from the U.S-based cohort were not reproduced in other cohorts. First, the prevalence and severity of obesity in these cohorts were lower relative to the United States which truncates the high end of the range of systemic leptin concentrations studied. This is an important limitation of these studies since the US-based study suggests a nonlinear or threshold effect for the highest quartile of serum leptin concentrations on asthma risk [66]. Further, the New Zealand cohort used nonfasting measures of serum leptin, resulting in nondifferential information bias in which the bias may contribute towards a lack of association due to increased variability [65]. Insufficient power or type II error is a possible explanation for the discrepant results as well. A sample size of approximately 407 women in the New Zealand study [65] and 1,446 young women in the Finland study [64] was smaller than that analyzed in the U.S.-based study [66]. 

#### 3.1.4. Clinic-Based Studies

The association between systemic leptin and asthma prevalence is more consistent in clinic-based studies of children than adults. In two case-control studies of prepubertal children by Guler et al. and Gurkan et al., serum leptin concentrations were higher in asthmatics than controls, independent of BMI [57, 60]. Additionally, Guler et al. found that this association was the strongest among atopic boys [57]. The study of Gurkan et al. also included mostly boys [60]. Similarly, in another study of peripubertal children, mostly boys, serum leptin concentrations in overweight asthmatics were twice that of controls of similar BMI (median values 30.8 versus 14.3 ng/mL) [58].

Data in adults from clinic-based studies are less convincing. Lessard et al. showed higher sputum leptin concentrations in adult asthmatics than controls, but these findings may be confounded by the remarkably different BMI levels between the groups [116]. While one small case-control study showed greater serum leptin concentrations among adults with asthma than controls (mean values 24.8 versus 13.7 ng/mL) [70], others failed to show any difference in serum or BAL leptin concentrations between the two groups, after adjusting for obesity [25, 36, 71, 72, 116]. Of note, the clinic-based studies had significantly smaller sample sizes than the population-based studies which may have contributed to discrepant findings due to inadequate power. 

It is unclear why the leptin-asthma association is more consistent among children than adults. A possible explanation may be that asthma in children may be a more uniform phenotype, whereas in adults it is a heterogeneous collection of phenotypes and that leptin may be differentially associated with one of the various asthma phenotypes. Indeed, the asthma phenotype in children is more likely to be atopic than that in adults. Atopic status may modify the leptin-asthma association—this is suggested by the study by Guler et al. [57] but has not been convincingly proven by a statistically significant test of interaction between leptin and atopy on asthma outcomes in any study. Finally, leptin concentrations in children are more purely reflective of BMI than in adults in the absence of the modifying effect of sex hormones. 

**Figure 5:**
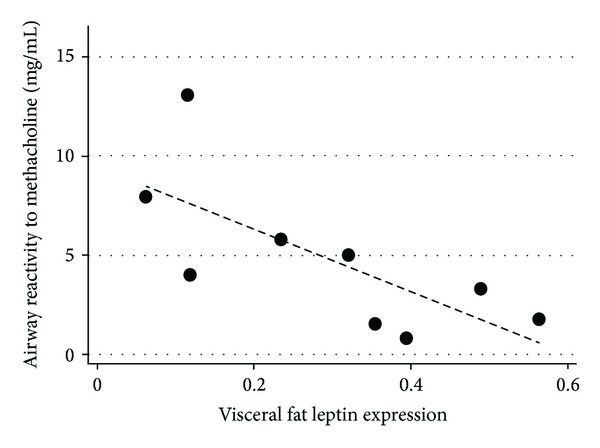
Relationship between visceral (omental) fat leptin expression and methacholine airway reactivity in morbidly obese women with asthma at the time of bariatric surgery (rho = −0.8; *P* = 0.001 for Spearman's correlation). Reproduced with permission of the American Thoracic Society. Copyright © 2013 American Thoracic Society. Official Journal of the American Thoracic Society [36].

In a small clinical study of obese women undergoing bariatric surgery, visceral (i.e., omental) adipose tissue from asthmatics showed greater expression of leptin than controls after adjustment for BMI, even though no differences in serum concentrations were reported [36]. Greater omental adipose tissue expression of leptin was also strongly related to airway hyperresponsiveness (i.e., lower methacholine PC_20_ values; Figure 5), while serum leptin concentrations were not similarly associated [36]. As discussed above, it is possible that leptin expression in omental adipose tissue is a marker for other adipose tissue dependent effects that modify asthma. 

There is also a recent human *ex vivo* cell-based study that supports a role for leptin in obesity-related asthma. Primary alveolar macrophages derived from overweight/obese adults with asthma generated greater levels of proinflammatory cytokines (such as IL-8 and TNF-*α*) after stimulation with leptin than macrophages derived from either normal weight asthmatics or obese nonasthmatics [117]. It is conceivable that this proinflammatory macrophage phenotype, in the context of high concentrations of serum leptin in obesity, may contribute to the pathogenesis of asthma associated with obesity [117]. 

*(ii) Asthma Severity*. Although still inconclusive, existing data suggest that high serum leptin concentrations are associated with greater asthma severity, particularly among prepubertal boys and peripubertal and postpubertal girls (Table 1). 

Clinic-based studies of prepubertal asthmatic children, mostly boys, showed that greater serum leptin concentrations were associated with greater clinical asthma severity [61], lower peak expiratory flow rates [60], greater severity of exercise induced bronchoconstriction [56], and higher serum total IgE concentrations [57]. Further, some studies have found these associations to be independent of BMI [56, 61]. In another study of peripubertal/postpubertal asthmatic subjects, Kattan et al. found a positive correlation between serum leptin and maximum asthma symptom days among girls. No such associations were found among boys, but sex interactions or BMI-adjusted results were not reported by the authors [62]. 

As was the case for asthma prevalence, the association between serum leptin and asthma severity is less consistent in adults than in children. A small case-control study showed that high serum leptin concentrations may discriminate women with severe asthma from mild/moderate asthma, although this association may be confounded by the remarkably different BMI values between the groups [70]. Although serum leptin was not correlated with airway inflammatory markers such as exhaled breath condensate-pH or exhaled nitric oxide (FeNO) levels among subjects with asthma, it was inversely correlated with FEV_1_/FVC ratio in the subgroup with mild/moderate asthma [70]. Another small clinic-based case-control study by Holguin et al. showed no associations between serum or BAL leptin and lung inflammatory biomarkers [25].

*(iii) Subgroup Effects*. Although the association between serum leptin and asthma prevalence or severity may be stronger and more consistent in specific population subgroups [57, 63, 66, 67, 118] such as prepubertal boys, peripubertal or postpubertal girls, and premenopausal women, the studies do not demonstrate a statistically significant sex, menopause, or age interaction. Similarly, significant interactions between serum leptin and atopy or smoking on asthma outcomes have not been reported. 

Even in studies demonstrating a leptin-asthma association, serum leptin does not appear to be the only intermediary factor that explains the obesity-asthma association. For example, Sood et al. [66] showed that the association between BMI and asthma in women was only slightly attenuated after adjustment for serum leptin concentration. Hence, other metabolic pathways and/or mechanical factors likely contribute to the obesity-asthma association. 

*(iv) Direction of Association*. In sensitized mice, allergen inhalation increases serum leptin concentrations [109]. Among humans with mild atopic asthma, transient bronchoprovocation from an experimental inhalational allergen challenge does not acutely affect serum leptin concentrations [75]. On the other hand, severe asthma exacerbations requiring hospitalization are associated with a transient increase in serum leptin concentrations [70]. As discussed above, hypoxemia consequent to bronchoconstriction or systemic spillover of airway inflammation in severe asthma exacerbations may increase secretion of leptin from adipose tissue [70]. Since there are no longitudinal studies examining the association between high serum leptin concentrations and human asthma, the temporal sequence for this association cannot be definitively established.

In summary, although leptin and its receptors are expressed in human airway cells, our understanding of the relationship between leptin and asthma is still evolving. Current evidence suggests that systemic leptin or visceral fat expression of leptin may be associated with greater asthma prevalence and/or severity, particularly among prepubertal boys, peripubertal and postpubertal girls, and women. It remains to be established whether modulation of leptin, independent of BMI, may be helpful in asthma prevention or treatment. 

## 4. Resistin 

Resistin (or “resistance to insulin”), a proinflammatory adipokine originally discovered in mice, was named for its ability to resist insulin action [119]. Resistin belongs to the RELM/FIZZ family that includes three additional cysteine-rich secretory proteins that share homology with resistin (called resistin-like molecules or RELM, namely, RELM-*α*, RELM-*β*, and RELM-*γ*). Studies on animals suggest that resistin and resistin-like molecules may induce inflammation, angiogenesis, and smooth muscle cell proliferation, all processes that are relevant to asthma pathogenesis [120, 121]. Interestingly, resistin induced vascular smooth muscle proliferation is inhibited by adiponectin [122], suggesting that not only the total concentrations but also the balance of pro- and anti-inflammatory adipokines may be important.

Much less data exists addressing a possible relationship between resistin and asthma than that existing for either leptin or adiponectin and asthma. Interestingly, human studies of resistin show associations opposite to those expected from *in vitro* studies of resistin. In a study of children by Kim et al., atopic asthmatics had lower resistin levels than nonatopic asthmatics and controls [55]. There was a negative correlation between serum resistin and eosinophil counts or serum total IgE and a positive correlation with methacholine PC_20_ (implying lower airway hyperresponsiveness) [55]. Multiple regression analysis revealed that after adjustment for other adiposity measures such as BMI and serum leptin and adiponectin, low serum resistin concentrations were still strongly predictive of asthma [55]. 

## 5. Summary

Although murine data are convincing, it is less clear whether adiponectin, leptin, or resistin plays a role in modulating asthma risk and/or severity in human subjects, although most of the human data are still limited to association studies. There are many possible explanations for the conflicting findings, as discussed above. Most of the human studies described above did not provide phenotypic characterization of asthmatics. In murine studies, there are opposing effects of adiponectin depending on whether the asthma model used was an allergic one or one related to oxidative stress. Hence, it is conceivable that adipokines may only be important for certain phenotypes of asthma. Indeed it is possible that the associations observed in certain subgroups (i.e., prepubertal boys) may relate to greater uniformity of asthma phenotypes within these populations. Finally, it is likely that adipokines are only one part of the obesity-asthma puzzle. Mechanical, developmental, hormonal, genetic, and epigenetic effects of obesity may also affect both asthma prevalence and severity in obese humans. 

## Figures and Tables

**Table 1:** A tabular summary of the current evidence supporting the roles for systemic adiponectin or leptin with respect to asthma in human subpopulations.

	Adiponectin-asthma association		Leptin-asthma association	
	Asthma prevalence	Asthma severity	Asthma prevalence	Asthma severity
Males				
Prepubertal boys	Inadequately studied (#)	Beneficial effect on exercise induced bronchoconstriction and FEF_25–75%_ [55, 56] (∗)	Harmful effect [57–59] (†)	Harmful effect on clinical outcomes; peak expiratory flow rates; exercise induced bronchoconstriction [56, 60, 61] (†)
Peri/postpubertal boys	No effect [55] (‡)	Beneficial effect on clinical outcomes and FEV_1_/FVC ratio [62] (∗)	No effect [55] (‡)	No effect [62] (‡)
Men	Unclear effect—no effect on clinical outcomes [63, 64]; harmful effect on prevalent reversible airflow obstruction; beneficial effect on exhaled nitric oxide [63, 65] (‡)	Harmful effect on clinical outcomes [49] (†)	No effect [64–66] (‡)	Inadequately studied (#)

Females				
Prepubertal girls	Inadequately studied (#)	Inadequately studied (#)	Inadequately studied (#)	Harmful effect on clinical outcomes [61] (†)
Peri/postpubertal girls	Beneficial effect [67] (∗)	Possible beneficial effect on clinical outcomes and spirometry [68] (∗)	Harmful effect [67] (†)	Harmful effect on clinical outcomes [62] (†)
Premenopausal women	Beneficial effect [36, 63, 69] (∗)	Beneficial effect on clinical outcomes [49] (∗)	Harmful effect [66] (†)	Inadequately studied (#)
Postmenopausal women	Inadequately studied (#)	Beneficial effect on clinical outcomes [70] (∗)	Harmful effect [70] (†)	Harmful effect on clinical outcomes and FEV_1_/FVC ratio [70] (†)

Note 1: Inadequately studied, beneficial; and harmful associations as well as no effects or unclear effects are depicted by different symbols (#, ∗, †, and ‡).

## Abbreviations

AMPK: Adenosine monophosphate-activated protein kinase
BAL: Bronchoalveolar lavage
BMI: Body mass index
COPD: Chronic obstructive pulmonary disease
ERK: Extracellular signal-regulated kinases
FeNO: Fractional excretion of nitric oxide
FEF_25–75%_: Maximum midexpiratory flow
FEV_1_: Forced expiratory volume in one second
FEV_1_/FVC: Ratio of forced expiratory volume in one second to forced vital capacity
HDL: High-density lipoprotein
IL: Interleukin
JAK: Janus-activated kinase
MAPK: Mitogen-activated protein kinase
NHANES III: Third National Health and Nutrition Examination Survey
OB-R: Leptin receptor
PI3K: Phosphatidylinositide 3-kinases
PC_20_: Provocative concentration of methacholine causing a 20% fall in FEV_1_
PDGF: Platelet-derived growth factor
PPAR: Peroxisome proliferator-activated receptor
PKB: Protein kinase B
RELM: Resistin-like molecule
S1P: Sphingosine-1-phosphate
SNP: Single-nucleotide polymorphism
STAT: Signal transducer and activator of transcription
TLR: Toll-like receptor
TNF: Tumor necrosis factor
VEGF: Vascular endothelial growth factor.
